# Regulation of CD8^+^ T memory and exhaustion by the mTOR signals

**DOI:** 10.1038/s41423-023-01064-3

**Published:** 2023-08-15

**Authors:** Yao Chen, Ziyang Xu, Hongxiang Sun, Xinxing Ouyang, Yuheng Han, Haihui Yu, Ningbo Wu, Yiting Xie, Bing Su

**Affiliations:** 1 Shanghai Institute of Immunology, Department of Immunology and Microbiology, and The Ministry of Education Key Laboratory of Cell Death and Differentiation, Shanghai Jiao Tong University School of Medicine, Shanghai, 200025 China; 2 Department of Tumor Biology, Shanghai Chest Hospital, Shanghai Jiao Tong University School of Medicine, Shanghai, 200025 China; 3 Center for Immune-Related Diseases at Shanghai Institute of Immunology, Department of Gastroenterology, Ruijin Hospital, Shanghai Jiao Tong University School of Medicine, Shanghai, 200025 China; 4 Shanghai Jiao Tong University School of Medicine–Yale Institute for Immune Metabolism, Shanghai Jiao Tong University School of Medicine, Shanghai, China; 5 Key Laboratory of Molecular Radiation Oncology of Hunan Province, Xiangya Hospital, Central South University, Changsha, China

**Keywords:** mTOR, Sin1, CD8^+^ T cell, T-cell memory, T-cell exhaustion, Signal transduction, Adaptive immunity

## Abstract

CD8^+^ T cells are the key executioners of the adaptive immune arm, which mediates antitumor and antiviral immunity. Naïve CD8^+^ T cells develop in the thymus and are quickly activated in the periphery after encountering a cognate antigen, which induces these cells to proliferate and differentiate into effector cells that fight the initial infection. Simultaneously, a fraction of these cells become long-lived memory CD8^+^ T cells that combat future infections. Notably, the generation and maintenance of memory cells is profoundly affected by various in vivo conditions, such as the mode of primary activation (e.g., acute vs. chronic immunization) or fluctuations in host metabolic, inflammatory, or aging factors. Therefore, many T cells may be lost or become exhausted and no longer functional. Complicated intracellular signaling pathways, transcription factors, epigenetic modifications, and metabolic processes are involved in this process. Therefore, understanding the cellular and molecular basis for the generation and fate of memory and exhausted CD8^+^ cells is central for harnessing cellular immunity. In this review, we focus on mammalian target of rapamycin (mTOR), particularly signaling mediated by mTOR complex (mTORC) 2 in memory and exhausted CD8^+^ T cells at the molecular level.

## Introduction

CD8^+^ T cells, commonly known as cytotoxic T lymphocytes, are critical for immune system-mediated defense against pathogens, including viruses and bacteria, as well as for tumor cell surveillance. In an acute infection, antigen-specific naïve CD8^+^ T cells are activated and differentiate into effector cells. After antigen clearance, 5–10% of these effector T cells become memory cells, which provide more robust and long-term protection against previously encountered pathogens [1]. However, in cancer and chronic infections where antigens persist, CD8^+^ T cells progressively differentiate toward a dysfunctional state of exhaustion [2]. An increasing number of studies have revealed that the pools of memory and exhausted CD8^+^ T cells are heterogeneous and comprise distinct subsets that vary in terms of their phenotype, function, and location within the body [3–13]. This heterogeneity reflects the complex nature of the immune system and the need for diverse and specialized immune cells to effectively respond to a wide range of pathogens and environmental challenges. However, our understanding of the molecular mechanisms and signaling pathways regulating the fate determination of CD8^+^ T cells is not yet complete.

**Fig. 1:**
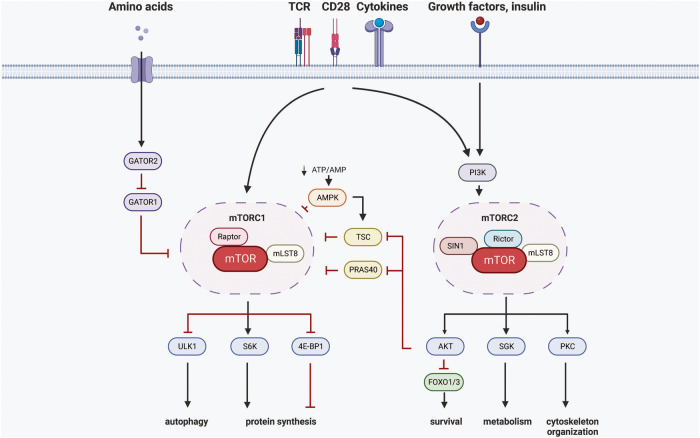
mTORC1/mTORC2 integrate diverse extracellular cues. Upon stimulation by amino acids, GATOR2 inhibits GATOR1, leading to a conformational change in Regulator. This change affects the GTP/GDP state of RagA/RagC and facilitates the recruitment of mTORC1 to the lysosome surface, where it becomes activated. Metabolic stress, such as low glucose, can inhibit mTORC1 through Rag-GTPase-dependent or AMPK-dependent mechanisms. Active AMPK directly phosphorylates Raptor or indirectly phosphorylates and activates TSC2 to inhibit mTORC1. Once mTORC1 is activated, downstream kinases S6K and translation initiation factor 4E-BP1 are phosphorylated, collectively promoting protein synthesis. To prevent futile metabolism, the autophagy activator ULK1 is phosphorylated by active mTORC1, thereby inhibiting autophagy initiation. On the other hand, mTORC2 has been primarily recognized as downstream integrators of insulin stimulation. Various immune-related signaling pathways, including TCR-signaling, CD28-mediated co-stimulatory signaling, and cytokines, have been identified to modulate mTORC2 activity. AGC kinase family members serve as the primary effectors for mTORC2. As a multifunctional kinase, active Akt phosphorylated by mTORC2 can also mediate mTORC1 activity by either blocking the inhibitory effects of the raptor-binding protein PRAS40 on mTORC1 or dissociating the TSC complex from the lysosomal surface, thereby enabling Rheb-mediated mTORC1 activation

The mammalian target of rapamycin (mTOR) was discovered in 1975 after rapamycin was isolated from *Streptomyces hygroscopicus*, a soil bacterium on Easter Island [14, 15]. mTOR signaling regulates a variety of cellular and molecular functions, including protein synthesis, cell growth and proliferation, autophagy, metabolism, and gene expression [16–19]. There are two distinct complexes of mTOR, mTOR complex (mTORC)1 and mTORC2, which share certain components, including the core kinase mTOR, mLST8 (also known as GβL), and DEPTOR [17]. However, each complex consists of unique components that contribute to its distinct functions and downstream signaling pathways. For example, regulatory-associated protein of mTOR (Raptor) is a specific component of mTORC1, while rapamycin-insensitive companion of TOR (Rictor) and Sty1/Spc1-interacting protein 1 (Sin1) are specific to mTORC2 [17, 20] (Fig. 1). mTORC1 is sensitive to rapamycin, and its activity is rapidly inhibited upon rapamycin treatment [21]. In contrast, mTORC2 is relatively resistant to rapamycin, and its activity is modestly inhibited after prolonged periods of treatment [22]. mTORC1 primarily regulates cell growth and metabolism, while mTORC2 is involved in cell survival and cytoskeletal organization [17]. In T cells, mTOR is activated by various signals, including growth factors, cytokines, T-cell receptor (TCR) stimulation, costimulatory signals, nutrient availability, and cellular energy status [23] (Fig. 1). Our previous studies showed that the Sin1 component of mTORC2 plays a critical role in various phases of T-cell functional maturation, including T-cell development, cytokine production, and immune niche regulation [24–26]. Overall, by functioning as a central hub that coordinates multiple signaling pathways, mTOR plays a critical role in regulating various aspects of T-cell function, including T-cell development, activation, differentiation, migration, survival, memory formation, and exhaustion. As the role of mTOR in regulating T-cell function has been previously discussed in multiple high-quality reviews [23, 27], this review focuses mainly on the Sin1/mTORC2 complex-mediated regulation of CD8^+^ T-cell fate decisions and the most recent studies that have reported previously unknown roles for mTOR signaling in T-cell memory and exhaustion generation.

### Heterogeneity and cell-fate decisions in effector and memory subsets

**Fig. 2:**
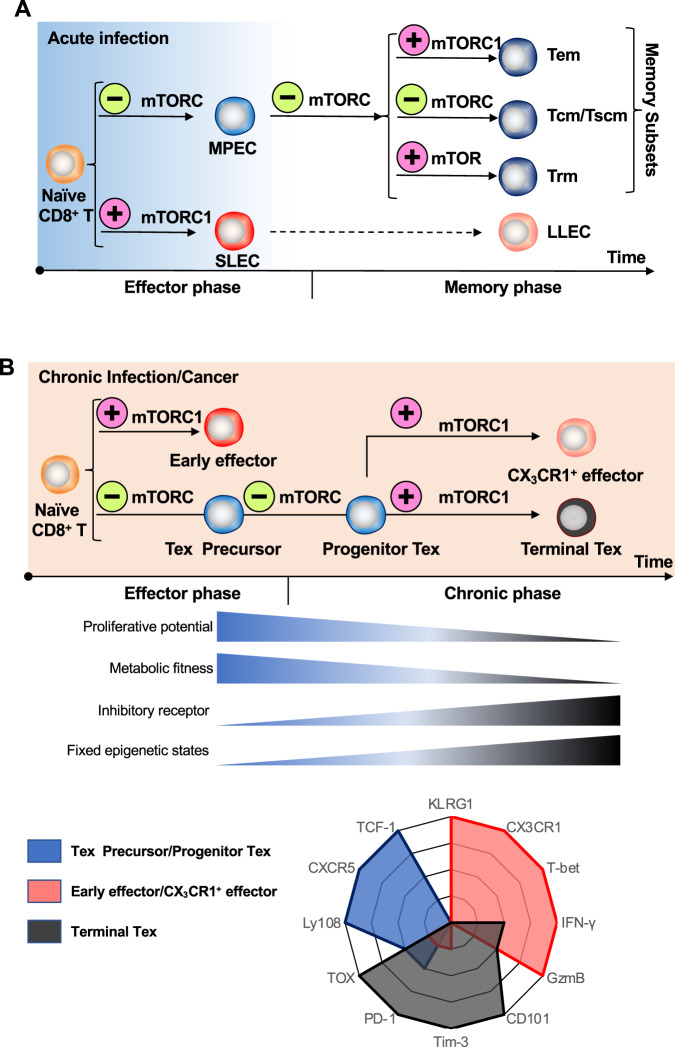
The role of the mTOR signaling pathway in the differentiation of memory/exhaustion T-cell subsets. **A** During acute infection, virus-specific naïve CD8^+^ T cells differentiate into SLECs that display potent cytotoxicity and MPECs that have a greater capacity to form memory cells following viral clearance. After the antigen has been eliminated, a minority of SLECs manage to survive and transform into LLECs. On the other hand, MPECs develop into various types of memory T cells, including Tem, Tcm, and Trm. mTORC1 activity instructs SLEC and Tem differentiation. Inhibition of mTOR, either by Rapamycin treatment or through siRNA-mediated knockdown, promotes MPEC and memory formation, particularly for Tcm and human Tscm. However, mTOR activation in T cells promotes their differentiation into Trm cells and enhances their survival in peripheral tissues. **B** During chronic infection or cancer, virus-specific naïve CD8^+^ T cells are activated and segregate into early effector cells and precursor cells. The precursor cells develop into Progenitor Tex, which further differentiate into terminally exhausted cells and effector-like cells marked by CX_3_CR1 expression. Inhibition of mTOR activity enhances Progenitor Tex formation at both the early and late stages. However, Progenitor Tex cells retain the ability to activate the mTOR pathway in response to antigen receptor signals, and mTOR is required for the transition of Progenitor Tex cells into exhausted and effector cells in the chronic phase. MPEC memory precursor effector cells, SLEC short-lived effector cells, LLEC long-lived effector cells, Tem effector memory T-cell, Tcm central memory T cells, Tscm T memory stem cells, Trm resident memory T cells, Progenitor Tex progenitor exhausted T-cell

During acute infection or vaccination, after priming by antigen-presenting cells in secondary organs, including the spleen and lymph nodes, antigen-experienced CD8^+^ T cells in the effector phase differentiate into two subsets with distinct features: the majority of these CD8^+^ T cells (~90–95%), termed short-lived effector cells (SLECs), are marked by high expression of CX_3_CR1 and KLRG1 and low expression of IL-7Ralpha (CD127) and the ability to actively clear virus-infected or transformed cells by secreting high amounts of perforin, granzyme and cytokines. The other CD8^+^ T cells (~5–10%), termed memory precursor effector cells (MPECs), are marked by low expression of CX_3_CR1 and KLRG1 and high expression of CD127 and are thought to give rise to the majority of memory T cells in the memory phase [28, 29] (Fig. 2A). Although MPECs exhibit several memory T-cell features, including high secretion of IL-2 after restimulation and high dependence on the memory/naïve T-associated transcription factor (TF) TCF-1 [28, 30, 31], MPECs and SLECs show comparable cytotoxic effector function [32]. During the initial expansion of CD8^+^ T cells, factors including TCR signals [33, 34], costimulatory/inhibitory molecules [35] and cytokines within the inflammatory environment [1, 36, 37] dictate the direction of SLEC/MPEC differentiation. TFs activated in each subset, including ZEB2, BLIMP1, and ID2 in SLEC [38–40] and ID3, FOXO1, and TCF-1 in MPECs [40, 41], contribute to the maintenance of subset identity in part by suppressing the expression of genes associated with the other subset.

When an antigen is cleared (the effector phase ends), most SLECs undergo apoptosis, but a small percentage of them survive to become long-lived effector cells (LLEs), which express high levels of CX_3_CR1 and KLRG1, intermediate levels of CD127 and low levels of Eomes during the memory phase [42, 43] (Fig. 2A). Despite inferior expansion ability after bacteria/virus rechallenge, these cells show superior clearance effectiveness of certain antigens by secreting cytolytic molecules, including GZMB and perforin [42, 43]. MPECs, on the other hand, differentiate into effector memory T cells (Tem), central memory T (Tcm) and residential memory T (Trm) cells [44–46] (Fig. 2A). Although high expression of CCR7 and CD62L enables Tcm cells to preferably localize to lymphoid tissues, lack of CCR7 and CD62L expression promotes Tem cells to take up residence in nonlymphoid tissues where they can be rapidly mobilized to sites of infection [47–49]. Tem cells, which also exhibit an immediate level of CX_3_CR1 expression and lack CD62L, shows better cytotoxic effector function but lower proliferative capacity than Tcm cells [50, 51]. Furthermore, a specific type of T-cell known as “T memory stem (Tscm) cells” is found in both human and mouse immune systems [52]. Tscm and Tcm cells both show high potential for self-renewal and produce Tem and effector cells after restimulation. However, Tscm cells are the least differentiated and thus the most immature memory T-cell subset and exhibit the highest self-renewal capacity [52]. They express high levels of CD95, CD122, and CD127 but do not express markers, such as CD27 and CD28, associated with effector and memory T-cell subsets [52].

To function in local immune defense and confer immediate protection against pathogenic infections at the site of initial infection, Trm cells are formed in peripheral tissues, such as skin, gut, lung, and other mucosal tissues, where they provide long-term protection against pathogenic infections. Although the true precursors of Trm cells are still under investigation, a fraction of KLRG1^-^ CD8^+^ T cells (presumably MPECs [53–57]) infiltrate tissues through chemokine–chemokine receptor interactions (such as the CXCL9/CXCL10–CXCR3 interaction) and form Trm cells [58]. Locally released cytokines, including IL-15, further upregulate the TF Hobit (*Zfp683*), which in synergy with Blimp1 controls a universal tissue-residency program and represses tissue-egressing genes, including S1PR1, CCR7 and KLF2, thereby promoting the formation of Trm cells [59]. A recent study, however, showed that at the protein level, the TFs BLIMP1 and ID3 controlled distinct Trm cell subsets within the small intestine: BLIMP1^hi^ Trm cells exhibit signatures consistent with SLECs (high KLRG1 and GZMB expression), and ID3^hi^ Trm cells exhibit signatures consistent with MPECs (high BCL2 and CD127 expression); this observation makes sense because the Blimp1^hi^ Trm cell population peaks early, while the number of ID3^hi^ Trm cells gradually increases after infection [60]. CD69 and CD103, two classic markers of Trm cells, are expressed at different levels in the two aforementioned Trm cell subsets [60]. In addition to intraorgan heterogeneity, Trm cells from different organs exhibit various levels of CD69 and CD103 expression [61], further suggesting interorgan heterogeneity. Indeed, the heterogeneity of Trm cells at the transcriptome/epigenome levels among tissues has been consistently observed [61–63]. Maintenance of these expression patterns by TGF-β signaling, which had been previously thought to be crucial for all Trm cells [58, 64, 65], is required only for Trm cells in the small intestine, salivary gland and skin, while Trm cells within fat tissue, kidney and liver are largely unaffected by the loss of TGF-β signaling [61, 63].

### Cellular and molecular mechanisms underlying T-cell exhaustion

Notably, during chronic infection or tumorigenesis, antigen-specific CD8^+^ T cells fail to transit into functional memory T cells. Unresolved chronic antigen stimulation altered costimulatory and coinhibitory signaling, and chronic inflammation cause CD8^+^ T cells to undergo a unique differentiation process commonly known as T-cell exhaustion [66–68]. T-cell exhaustion is characterized by progressive loss of effector function, including cytotoxicity and cytokine production ability. For example, exhausted cells have been reported to produce lower levels of cytokines, including IL-2, tumor necrosis factor (TNF), and interferon gamma (IFN-γ), and fewer cytotoxic effector molecules, including perforin and granzymes [66]. Another key feature of T-cell exhaustion is the evaluated expression of multiple inhibitory receptors (IRs), including PD1, TIM3, CTLA-4, LAG3, TIGIT, CD160, BTLA, and 2B4 (CD244) [69]. Notably, using monoclonal antibodies that target IRs to enhance T-cell function has been shown to be a promising treatment for cancer patients.

Persistent T-cell exposure to antigens is critical in driving T-cell exhaustion and is a key condition induced by various chronic infections and cancers both in model mice and humans [66]. Both high antigen levels and long-term antigen persistence drive T-cell exhaustion. Previous studies revealed that infections associated with high levels of persistence viremia, including viremia induced by human immunodeficiency virus (HIV), hepatitis C virus (HCV), hepatitis B virus (HBV), and mouse lymphocytic choriomeningitis virus (LCMV), induce more severe T-cell dysfunction [70–73]. In contrast, infections such as cytomegalovirus (CMV) and Epstein–Barr virus (EBV), which induce chronic infection but low levels of viremia, have been shown to induce an intermediate rate of T-cell dysfunction [74]. In HIV patients, restored functional antiviral CD8^+^ T cells are found when viral levels decline after successful antiretroviral therapy (ART) [75, 76]. Furthermore, antigen at high levels has been reported to directly drive T-cell exhaustion during chronic infection and is not a consequence of T-cell exhaustion [77]. In addition to antigen load, sustained antigen exposure leads to T-cell exhaustion. Acute viral infections that resolve in a short period leads to the initiation of strong effector and durable memory responses. However, antigen-specific CD8^+^ T cells adoptively transferred from the late phase of chronic infection to uninfected mice failed to develop into fully functional cytotoxic T cells (CTLs) or memory T cells [78, 79]. Indeed, long-term antigen exposure irreversibly modifies the metabolic, transcriptional, and epigenetic programs underlying T-cell function [80–82].

Decreased costimulatory and increased coinhibitory signals promote T-cell exhaustion. As discussed above, exhausted CD8^+^ T cells express multiple IRs at persistent levels, and ligands related to these IRs are upregulated on antigen-presenting cells (APCs), tumor cells, and nonimmune cells during chronic infections and in cancer. Notably, PD-1 is transiently upregulated during T-cell activation and is required for memory formation [83]. In the contexts of chronic infections and cancer, the expression of PD-1 is maintained at high levels on exhausted cells, which suppresses their effector function. Mechanistically, after PD-1 and PD-L1 ligation, SHP-2 is activated and dampens signaling pathway activation induced after TCR and CD28 stimulation [84, 85]. Although the PD-1 pathway is a critical mediator of T-cell exhaustion, some studies reported that exhausted T (Tex) cells are developed under PD-1-abrogated conditions, indicating that PD-1 does not directly cause T-cell exhaustion [86]. Furthermore, PD-1^−/−^ CD8^+^ T cells exhibit a tendency to undergo more pronounced exhaustion [86]. These reports suggest that TCR stimulation is the major cause of T-cell exhaustion and that PD-1 deficiency results in stronger TCR signaling that drives a more severe exhaustion phenotype. In addition to PD-1, other IRs, such as Lag-3, CD160, Tim-3, CTLA-4, and TIGHT, have all been shown to suppress T-cell function and are highly expressed by exhausted CD8^+^ T cells [87]. The CD28 pathway is an essential costimulatory pathway in CD8^+^ T cells, and CD28 ligand binding reduces the TCR signaling threshold required for T-cell activation [88]. CD28 signaling is impaired in cases of chronic infection and cancer [89]. PD-1 dephosphorylates CD28, and CD28 stimulation is essential for potent anti-PD-1 therapy during chronic viral infection in mice [89]. In addition to dephosphorylation by PD-1, CTLA-4 at high levels in the chronic infection or cancer context competes for the ligands of CD28, namely, B7 family receptors, thereby diminishing CD28 signaling [66].

During acute viral infection, a proinflammatory environment is required for both the generation and function of effector CD8^+^ T cells, while memory cell formation requires a less pronounced proinflammatory environment [1]. However, a highly proinflammatory environment in the context of chronic infection or cancer can result in the induction of suppressive cytokines that promote T-cell exhaustion. Type I interferons are antiviral cytokines, but IFN-α/β at elevated levels has been reported to prompt the expression of negative regulators such as IL-10 and PD-L1, which promote T-cell exhaustion [66]. IL-10 and TGF-β are abundance in cases of chronic infections or cancer, and attenuation of IL-10 and TGF-β signaling contributes to the prevention and reversal of T-cell exhaustion [90–92].

T-cell exhaustion is associated with an altered metabolic program. During chronic infection, IRs induce signaling events that suppress Akt activation and mTOR activity in Tex cells, which leads to reduced glucose uptake, repressed cellular respiration, and dysregulated mitochondrial energetics [93]. In the tumor microenvironment, tumor cells compete with immune cells for fuel sources, such as glucose and oxygen, which may contribute to the metabolic reprogramming of T cells and promote their exhaustion [94]. Furthermore, distinct transcriptional and epigenetic programs are acquired during the development of T-cell exhaustion [66]. Overall, Tex cells constitute a unique CD8^+^ subset that phenotypically and mechanistically differ from the effector and memory CD8^+^ T cells that are generated during acute infection.

The diversity of the mechanisms that lead to T-cell exhaustion highlights the challenge of developing therapeutic strategies. Specifically, the optimal combination therapies might target different signaling pathways to achieve better and more durable clinical outcomes in the treatment of chronic infection and cancer.

### Heterogeneity and differentiation trajectories of exhausted CD8^+^ T cells

Previously, antigen-specific CD8^+^ T cells during chronic infection and cancer were collectively termed Tex cells. Recently, multiple studies have shown that the pool of exhausted CD8^+^ T cells is heterogeneous and comprises several phenotypically and functionally distinct subsets. During the onset of chronic infection or tumor growth, although some antigen-experienced activated CD8^+^ T cells exhibit features of early effectors (KLRG1^Hi^CX_3_CR1^+^PD-1^lo^TCF-1^lo^), the others become progenitor exhausted T cells (progenitor Tex cells) marked by high expression of Ly108, intermediate level expression of PD-1 and low expression of CX_3_CR1 and KLRG1 [3, 95]. Governed by the master TF TCF-1, progenitor Tex cells are quiescent but show the highest capacity to proliferate and give rise to other Tex cell subsets [4, 7, 8, 96, 97]. These unique features enable progenitor Tex cells to expand robustly during immunotherapy treatment [5, 7]. In accordance with the role of TCF-1 in establishing progenitor Tex cells, loss of this TF leads to the abrogation of the exhausted T-cell lineage, with the formation of the aforementioned early effector cells largely unaffected [3]. Recent studies based on single-cell technologies have revealed functional and spatial heterogeneity within the progenitor Tex cell subset: in chronic infections, a precursor subset marked by high expression of CD62L [98] or CD69 [99] is thought to give rise to progenitor Tex cells, while stem-like CD8^+^ T cells residing in draining lymph nodes may be precursors of progenitor Tex cells in the tumor microenvironment [100–102] (Fig. 2B).

At later stages of chronic infection or tumor growth, when CD4^+^ T-cell help is lost, continuous TCR signaling activates several downstream TFs, including TOX [10, 103, 104], NR4A [105, 106], NFAT [107, 108] and IRF4 [109], which in conjunction boost the differentiation of progenitor Tex cells into Tex cells. Gradually increased expression of inhibitory receptors, including PD-1, TIM-3 and CD101, is typical during this process [9, 110]. However, facilitated by CD4^+^ T-cell action (mainly their secretion of IL-21), progenitor Tex cells differentiate into a subset that exhibits higher cytotoxicity and cytokine production ability (denoted as CX_3_CR1^+^ effector T cells) [13, 111, 112]. This subset mirrors to some extent the SLEC subset that appears during acute infection due to their high expression of CX_3_CR1, KLRG1 and the TF T-bet [12, 13, 113]. Higher activity of the TF BATF in this cell subset is thought to maintain the cytotoxic program by direct binding to the *Tbx21* (encodes T-bet) and *Klf2* gene loci [12] (Fig. 2B).

### Transcriptional and epigenetic regulation of T-cell exhaustion

T-bet and Eomes both belong to the T-box TF family. In cases of acute infection, T-bet drives CTL differentiation, while Eomes promotes memory T-cell development [114]. During chronic infection, these two TFs are both needed to maintain the antigen-specific T-cell pool [95]. T-bet is specifically needed for CX_3_CR1^+^ effector T-cell formation in the chronic infection context [12]. However, Eomes plays a more complex role in regulating effector- and exhaustion-related genes; notably, it coordinates with T-bet to mediate functional effector differentiation, but it expressed at the highest level in terminally exhausted cells and promotes T-cell exhaustion in cases of chronic infection and cancer [13, 95, 115].

TCF-1 is critical for establishing CD8^+^ T-cell identity during T-cell development [116] and promotes the memory CD8^+^ T-cell response during acute infection [31, 117]. Importantly, TCF-1^+^ CD8^+^ T cells are progenitor Tex cells during chronic infection and cancer, and they survive long term, undergo self-renewal and retain the potential to produce differentiated cell subsets [7]. Similar to its role in programming memory cells after acute infection, TCF-1 has been reported to suppress effector cell differentiation during the early stage of chronic infection, possibly by promoting the expression of Eomes, which antagonizes the activity of T-bet and thus regulates the formation of progenitor cells [3].

Sustained expression of FOXO1 maintains T cells in a quiescent state, which is required for memory formation in the context of acute viral infection [41, 118], progenitor Tex cell in the context of chronic viral infection [119], and acquisition of the senescent phenotype by naïve T cells during aging [120]. The mechanism is, in part, constituted by direct FOXO1 binding and suppressing AP-1 TFs, which are key regulators of effector programs [120]. Moreover, FOXO1 drives the expression of TCF-1, a regulator central to memory programming in CD8^+^ T cells [41]. Furthermore, FOXO1 has been reported to control T-cell trafficking by directly inducing the expression of chemokine receptors such as CCR7 and S1P1 or by inducing KLF2 expression, which subsequently regulates the expression of CD62L and S1PR1 [23].

TOX is not required for the formation of effector or memory cells during acute infection [104]. Notably, several groups have reported that TOX is selectively upregulated during chronic infection and in cancer, and it transcriptionally and epigenetically programs T cells to undergo exhaustion [103]. The induction of TOX activity requires calcium signaling and NFAT2 [103], the activation of which is initially induced via prolonged TCR stimulation.

NR4A TF family members are located downstream of the TCR signaling pathway and are known to play a key role during thymocyte selection. The three TFs that constitute this family, NR4A1, NR4A2, and NR4A3, exhibit redundancy [121]. NR4A TFs have been reported to program CD8^+^ T-cell exhaustion in cases of chronic infection and cancer. A recent study further defined a role for TOX and the NR4A TF, which form a transcriptional network that mediates CD8^+^ T-cell exhaustion [122]. They act downstream of NFAT and form a feedback loop in which TOX and NR4A positively regulate each other to induce the transcriptional program that leads to T-cell exhaustion [122].

Epigenetic profiling revealed several fundamental discoveries of T-cell exhaustion. First, differentiation programs in exhausted CD8^+^ T cells are epigenetically imprinted after these cells are primed [123, 124]. The programs can be transiently reinvigorated, such as via PD-1 blockade, but fixed epigenetic landscapes irrevocably commit T cells to exhaustion [124]. Furthermore, a particular epigenetic landscape of Tex cells contributes to the unique TF networks and gene expression patterns of T-cell exhaustion [82, 125]. Some studies have attempted to epigenetically reprogram Tex cells. For example, immune functions were reestablished after diacetylated histone H3 levels were restored using a histone deacetylase inhibitor [126]. In addition, blocking de novo methylation-enhanced PD-1 blockade mediated T-cell reinvigoration [123]. These findings suggest that better clinical outcomes for T-cell-based immune therapies may be achieved by manipulating epigenetic programs (Fig. 2B).

### Metabolic dynamics instruct CD8^+^ T-cell differentiation

Naïve CD8^+^ T cells are maintained in a quiescent state, which requires relatively low energy as these primarily rely on oxidative phosphorylation (OXPHOS) in the mitochondria. When these cells are activated, the metabolism process undergoes reprogramming, and both aerobic glycolysis and OXPHOS pathways are then triggered to meet the high energy needed for rapid cell growth and proliferation [127]. In general, TCR stimulation induces signaling via the ERK/MAPK pathway and calcium influx, while CD28 signaling triggers the activation of the PI3K-Akt-mTOR axis [128, 129]. The two pathways collectively activate the NF-κB pathway [128, 129]. Furthermore, IL-2 and TCR have been implicated in activating PI3K-Akt-mTOR signaling [130]. After TCR stimulation, CD8^+^ T cells actively engage and rely on glycolysis and glutaminolysis driven by TFs such as c-MYC [131]. The PI3K-Akt-mTOR axis and MYC signaling are the primary regulators of early metabolic changes associated with T-cell activation and differentiation. The metabolic heterogeneity of CD8^+^ T cells was first observed between effector and memory T cells, and it is generally acknowledged that effector CD8^+^ T cells primary undergo aerobic glycolysis, whereas memory CD8^+^ T cells show enhanced OXPHOS and fatty acid oxidation activity [132, 133]. In line with the increased survival potential of memory cells, enhanced spare respiratory capacity (SRC, an indicator of how close to its bioenergetic limit a cell is functioning) fueled by fatty acid oxidation is critical for the formation as well as the recall response of memory cells [134, 135], and defects in fatty acid oxidation can impair memory T-cell formation [136]. Importantly, the mitochondrial metabolism underlying memory CD8^+^ T-cell function is unique in that it is a futile metabolic cycle triggered first by triacylglycerol (TAG) production from glucose and then the oxidization of TAGs into fatty acids [137]. Other ancillary metabolism pathways, including the pentose phosphate, cholesterol synthesis, polyamine synthesis and hexosamine synthesis pathways, have also been suggested to play important roles in T-cell functions, and their functions have been reviewed elsewhere [138].

Although no direct comparison of metabolic activity between SLECs and MPECs has been reported, one study showed that by interacting with IL-7Ralpha and further upregulating the glycerol channel aquaporin 9, IL-7 enables the import of glycerol and synthesis of triglycerides, which are critical for CD8^+^ T-cell maintenance of long-term metabolic fitness [139]. These metabolic advantages may have already been realized by the time that MPECs express IL-7Ralpha at higher levels than those expressed by SLECs, suggesting that metabolic heterogeneity among CD8^+^ T cells during the effector phase might dictate their future fates.

Paradoxically, although glycolysis is more often related to the effector function of CD8^+^ T cells, constitutive activation of the glycolysis pathway (together with loss of SRC) by knocking out VHL promoted MPEC formation, while memory-related features, including long-term maintenance, the recall response and virus clearance ability, were not hindered [140]. Interestingly, most of the memory CD8^+^ T cells produced after genetic manipulation exhibited features of Tem cells, and a direct comparison between Tem and Tcm cells confirmed preferential glycolysis in Tem cells, suggesting that altered metabolism skewed the contents in the heterogeneous memory pool [140]. This heightened glycolytic capacity allowed the Tem cells to proliferate at a faster rate than the Tcm or naïve T cells under hypoxic conditions [141].

Located within tissues where the oxygen level, nutrients availability and pH values may differ from those in the lymph node/circulation system, Trm shows evident metabolic adaptations [142–149]. Barrier tissues, where Trm cells were first described, are known to exhibit relatively hypoxic conditions [142, 143]. Notably, the combination of hypoxia and TGF-β could induce Trm-like CD8^+^ T cells in vitro [144]. Regarding nutrients availability, Trm cells in skin have been reported to show upregulated expression of fatty acid-binding proteins, including FABP4/FABP5, to leverage the oxidative metabolism of exogeneous fatty acids, allowing long-term survival in skin [145]. The selective upregulation of fatty acid-binding proteins was later shown to be an organ-specific feature [146]. Additionally, the activation status of Trm cells located in the epithelial barriers of the intestine is directly influenced by the availability of local metabolites, which stands in stark contrast to circulating CD8^+^ T cells [147, 148]. This effect is particularly pronounced in relation to glucose availability within the local environment [147, 148]. Although the role of pH sensing in CD8^+^ Trm cells is not clear, mutation of the pH-sensing protein GPR65 could potentially result in altered cellular metabolism of Th17/Th22 in the colon [149].

During chronic infection or tumorigenesis, the major metabolic pathways, including glycolysis and OXPHOS, in exhausted CD8^+^ T cells are severely impaired, although the precise nature of the deficiency depends partly on the disease [150]. For example, Tex cells highly depend on glycolysis to meet energetic needs and show poor capacity to perform optimal glycolysis compared to that of SLECs [151, 152]. Regarding mitochondrion-related metabolism, an early study indicated that at the early stage of chronic infection, the mitochondria of Tex cells exhibited higher mass but lower membrane potential, and this dysfunctional state was maintained throughout the late stage of the chronic infection [152]. Several studies later further substantiated the pivotal role of mitochondrial dysfunction in promoting T-cell exhaustion and revealed potential regulatory factors, including hypoxia, persistent antigen exposure, and IRs [153–155]. Consistent with these findings, strategies to increase the metabolic fitness of Tex cells have shown promising results for reinvigorating cell functionality.

### mTOR: an integrator of cellular metabolism sensors

Optimal T-cell survival and quick responses to stimulation are tightly controlled by metabolic alterations such as catabolism during energy generation and anabolism during biosynthesis [156]. One of the core regulators of metabolic reprogramming is the mTOR signaling cascade [157]. mTOR signaling controls the expression or functional activity of multiple metabolic enzymes by sensing alterations in metabolite levels, especially glucose and amino acid levels [158]. mTOR was first identified as the molecular target of the antifungal agent rapamycin discovered on Easter Island [14, 15, 159]. Over several decades of research, mTOR has been characterized as a key regulator of multiple metabolic processes, including ribosome biogenesis [160], protein [161]/nucleotide [162, 163]/fatty acid/lipid [164] synthesis and negative regulation of autophagy [165].

**Fig. 3:**
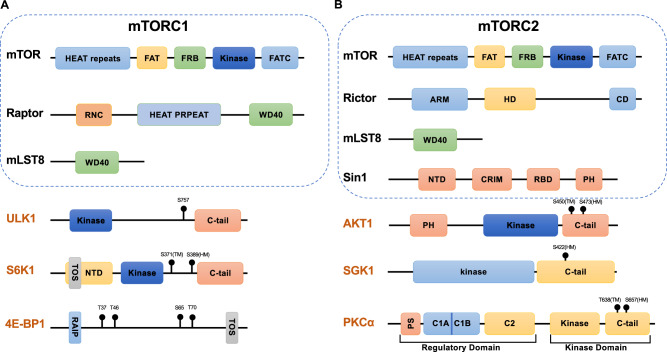
Composition of mTORC1/2 core subunits and selected substrates. **A** Main protein domains and phosphorylation sites of mTORC1 core subunits and selected substrates. mTOR and mLST8 are shared subunits of mTORC1/2, while Raptor and Rictor/Sin1 are the defining subunits for mTORC1 and mTORC2, respectively. S6K is phosphorylated by mTORC1 at S371 (TM) and T389 (HM) in the linker region. 4E-BP utilizes the TOS and RAIP motif to interact with Raptor/mTORC1 and is sequentially phosphorylated by mTORC1 at T37/T46 and S65/T70. ULK1 can be phosphorylated on S757. **B** Main protein domains and phosphorylation sites of mTORC2 core subunits and selected substrates. MLST8 interacts with Sin1 to position its substrate-interacting CRIM domain, providing substrate specificity of mTORC2. Sin1/mTORC2 phosphorylates T450 (turn motif) and S473 (hydrophobic motif) in the C-tail of Akt1. This dual phosphorylation has also been observed in PKC, while for SGK1, S422(HM) is the only known site phosphorylated by mTORC2. HEAT repeat found in Huntingtin, elongation factor 3 (EF3), protein phosphatase 2A (PP2A), and the yeast kinase TOR1, FAT FAK focal adhesion targeting, FRB FKBP-rapamycin-binding, FATC FRAP-ATM-TRRAP-C-terminal, RNC Raptor N-terminal conserved, NTD N-terminal domain, TOS TOR signaling, RAIP Arg-Ala-Ile-Pro motif, ARM Armadillo, HD HEAT-like domain, CD C-terminal domain, CRIM conserved region in the middle, RBD Ras-binding domain, PH Pleckstrin homology, PS pseudosubstrate, C1,C2 membrane targeting module

mTOR forms at least two distinct protein complexes localized in two different subcellular localizations, termed mTORC1 [166, 167] and mTORC2 [168, 169] (Fig. 1). mTORC1 is sensitive to rapamycin treatment or nutrient deprivation, while mTORC2 reacts only to growth factors [170]. mTORC1 is localized mainly on the surface of lysosomes [171]; the complex consists of mTOR, Raptor, mLST8, PRAS40 and DEPTOR (Fig. 3A). The physiological stimuli of mTORC1 are mainly nutrients, including amino acids [172, 173] and glucose. Amino acids (arginine, leucine) regulate the activity of four small GTPases called RagA/B/C/D [174], allowing the translocation of mTORC1 to the surface of lysosomes and affecting the phosphorylation of S6K and 4EBP1, which mediate optimal translational control. On the other hand, cellular homeostasis indicators, such as indicators of redox status, can regulate the activity of mTORC1 [175–177]; these indicators include DEPDC5, NPRL2 and NPRL3. GATOR1 is inhibited by GATOR2 [178], and the latter can be inhibited by the leucine sensor SESTRIN2 [179–181] and arginine sensor CASTOR [182]. Methionine-derived SAM, on the other hand, targets SAMTOR to activate mTORC1 [183].

mTORC2, on the other hand, is localized on mitochondrion-associated endoplasmic reticulum membrane [184]. mTORC2 contains core component of Rictor, Sin1/MAPKAP1 and PRR5, in addition to two core components of both mTOR complexes, mLST8 and mTOR (Fig. 3B). The integrity and activity of mTORC2 depends greatly on the close contact between Rictor and Sin1. The Sin1 N-terminal domain is embedded within Rictor and then folds around mLST8 [185], and this N-terminal domain undergoes another conformational change after substrate binding [186]. A conserved region in the middle (CRIM) of Sin1 is important for Sin1 substrate recruitment because it interacts with mLST8, and the CRIM domain has been verified to interact directly with SGK1, PKC and Akt [187]. After growth factor stimulation-induced PI3K activation, PIP3 facilitates the release of the Sin1 PH domain from autoinhibited mTORC2 and recruits mTORC2 to the cell membrane [188]. The hydrophobic motif (HM) of multiple AGC family kinases, such as Akt S473, is phosphorylated and triggers other downstream signaling cascades, such as FOXO nuclear export and GSK3 phosphorylation pathways, to promote cell survival. In addition, active Akt phosphorylates the mTORC2 component Sin1 at the T86 site, resulting in the establishment of a positive feedback loop [189, 190]. Moreover, mTORC2 shows the capability of phosphorylating another evolutionarily conserved site in AGC kinases in the turn motif at T450 of Akt [191, 192]. During Akt translation, mTORC2 is recruited to active ribosomes and phosphorylates newly synthesized Akt peptides. This phosphorylation event is critical for the correct protein folding and stabilization of Akt because it prevents co-translational ubiquitination and degradation. Recently, Baffi et al. identified a novel set of mTORC2 phosphorylation sites in a conserved motif: FXXXFT, called the TIM motif [193]. Their experiment suggested that in contrast to direct phosphorylation of HM sites, mTORC2 first phosphorylates TIM and then promotes T-loop phosphorylation and HM site autophosphorylation, relieving the nascent dimerization of AGC kinases such as PKC. In addition to its canonical AGC kinase substrates, mTORC2 has also been reported to phosphorylate multiple Hippo pathway components, including AMOTL2, MST1 and YAP [194–196], indicating that these two pathways synergistically control organ size by harmonizing cell size and cell number.

### Sin1-mediated mTOR signaling

In addition to the different biochemical functions and upstream regulatory effects of the two mTOR complexes, they regulate each other via multiple mechanisms. First, mTORC1 is negatively regulated by its downstream substrate S6K via the phosphorylation of IRS1 and reduction in the IRS1 protein level. This process downregulates insulin-PI3K signaling and thus inactivates mTORC2 [197]. Another known mechanism involves the phosphorylation of growth factor bound-receptor protein 10 (Grb10) at multiple sites and the subsequent stabilization of the protein. Grb10 inhibits the phosphorylation of the insulin receptors InsR and IRS1/2 and destabilizes IRS1, thus suppressing PI3K signaling and mTORC2 activity. S6K has also been reported to phosphorylate Rictor at Thr1135 to suppress mTORC2 formation.

mTORC2, on the other hand, due to its function as a kinase for Akt, inhibits the activity of the TSC complex and thus augments mTORC1 activity and triggers optimal growth factor-induced cell growth [198]. However, our study experiment with Sin1-knockout mouse embryonic fibroblasts demonstrated that Sin1 deficiency leads to the upregulated phosphorylation of S6K [22]. This outcome indicated a previously unknown role for mTORC2 as a potential negative regulator of mTORC1. Two explanations have been attributed to this phenomenon. First, the overall protein levels of mTOR complex subunits are quite low, especially the levels of the two core components mTOR and mLST8. Therefore, in Sin1-deficient cells, all the available mTOR may be integrated into the assembled mTORC1 complex. Another potential mechanism involves the mTORC2 downstream substrate FOXO1, which inhibits transcription of the mTORC1 suppressor Sestrin3 [199]. In either case, mTORC2 potentially inhibits mTORC1 activity. In addition, we recently found that the phosphorylation of one component of GATOR1 was altered in Sin1-deficient cells [20]. Although the detailed molecular mechanism is still unclear, we believe that the Sin1/mTORC2 complex may directly regulate the GATOR1-KICSTOR complex to regulate mTORC1 activity and control cell growth, metabolism, immune responses and tumorigenesis [20]. Thorough investigation into this molecular event will provide new insights into the mTORC1–mTORC2 interaction in addition to the mTORC2/Akt/TSC axis and possibly lead to the characterization of novel targets that can be disturbed for fine tuning mTOR-mediated metabolic regulation.

In addition to the reciprocal regulation between mTORC1 and mTORC2, these complexes may be activated synergistically. For example, a Ras homolog enriched in the striatum has been shown to interact with both complexes and activate mTOR activity [200]. PRAS40, on the other hand, interacts with both mTORC1 and mTORC2 and suppresses their activity [201]. Additionally, the small GTPase Rac1 promotes the activity of both complexes. Activation of the two mTOR complexes leads to the upregulation of both transcription and translation mediated through the mTORC1/S6K and mTORC2/Akt axes [202].

In summary, understanding the detailed molecular events that interlink mTORC1 and mTORC2 will advance the understanding of how eukaryotic cells integrate surrounding nutrient and growth factor signaling to coordinate optimal protein synthesis and cellular metabolism; this understanding can be then leveraged for the treatment of various metabolic diseases and promotion of healthy aging. For T cells, sensitivity to environmental cues and rapid responses are pivotal for the quick modulation of intracellular signaling and the execution of optimal effector functioning and immunological memory.

### mTORC1/mTORC2 activity differentially dictates CD8^+^ T-cell fate

During the effector phase, treatment with rapamycin leads to the acquisition of the MPEC phenotype by antigen-specific CD8^+^ T cells [203]. The result of this process is largely due to the reduced apoptosis rate of antigen-specific CD8^+^ T cells, which ensures the survival and differentiation of memory precursors into long-lived memory cells. Treatment with rapamycin during the memory phase leads to the significant enrichment of the CD127^+^CD62L^+^ Tcm cell population, and overall enhanced recall expansion and homeostatic proliferation are also observed [203]. This systemic rapamycin treatment-induced phenomenon has been further proven to be T-cell intrinsic and mTORC1 specific [203]. Further perturbation of CD8^+^ T-cell-specific Raptor or mTOR activity resulted in a phenotype similar to that observed in rapamycin-treated organisms, indicating T-cell-intrinsic mTORC1 suppression of memory T-cell differentiation [204, 205]. Consistent with this finding, loss of mTORC1 activity by genetically knocking out Rheb in T cells led to a higher CD127 level as well as accelerated formation of antigen-specific CD8^+^ T cells during the memory phase, although these cells failed to respond to further rechallenge [206]. Constitutive activation of mTORC1 by genetic knock out of TSC1 and TSC2 led to accelerated effector cell formation, as exemplified by higher KLRG1 expression, greater stimulation-induced expansion and increased cytokine production, but the memory function was severely impaired in these CD8^+^ T cells [206, 207].

The activity of the mTORC2 pathway also contributes to T-cell fate decisions. Loss of mTORC2 activity by Rictor knockout promoted the formation of MPECs and long-term antigen-specific memory T cells with better cytokine-producing ability after rechallenge [206]. Similar observations were subsequently reported, and Rictor deficiency led to significantly higher IL-2 production at the peak of the primary T-cell response [208]. The unphosphorylated form of FOXO1, established due to deficient mTORC2 activity (and further dampened SGK1 activity [209]), as been suggested to accumulate in the nucleus, where it promotes the expression of the memory-related TFs Eomes and TCF-1, while the expression of the effector-related TF T-bet was decreased, collectively leading to memory T-cell subsets formation [208].

mTORC1/2 governs the formation of Trm cells. Treatment with rapamycin or mTOR knockdown reduced the rate of memory CD8^+^ T-cell accumulation in both mesenteric lymph nodes and the small intestine after acute vesicular stomatitis (VSV) infection and concurrently reduced the expression of CCR9, Integrin alpha-4 and CD103, suggesting that memory CD8^+^ T-cell trafficking to the intestinal mucosa requires mTORC1 activity [210]. The accumulation of resident memory CD8^+^ T cells in the lung after influenza infection has also been reported to depend on mTORC1 activity [211]. Further analysis revealed that mTORC1 is located downstream of IL-15 and TGF-β and is critical for the upregulation of SLEC-/Trm-related TFs (such as BCL-6, T-bet and BLIMP-1), the increase in cytotoxic molecules (such as GZMB) and increased mitochondrial fitness [212]. Since IL-15 and TGF-β also enhance the phosphorylation of AktS473 and downstream phosphorylation of FOXO1 S253 [212], mTORC2 may participate in the formation of Trm cells via the action of factors downstream of the IL-15/mTORC1 pathway. Indeed, the tissue-egression program activity represented by the expression of Klf2, which is controlled by unphosphorylated for FOXO1, was diminished in the presence of IL-15/TGF-β [212].

During the process of T-cell exhaustion, CD8^+^ T cells in general exhibit impaired mTORC1 (measured by the phosphorylation rate of S6) and mTORC2 (measured by the phosphorylation rates of Akt S473 and FOXO1/3a) activity compared with that in antigen-specific T cells in the context of acute infection [213]. While mTORC1 activity was required for restored metabolism in Tex cells and enhanced their cytokine secretion after anti-PD-1 treatment, the suppressed mTORC2 activity sustained the FOXO1 in the unphosphorylated state, which is critical for upregulated PD-1 expression in Tex cells (later found to be essential for the formation of the Tex cell lineage), the T-bet^hi^ subset of Tex cells differentiated into the Eomes^hi^ cells and survived throughout chronic infection [213]; these data support a “maladaptation” view of T-cell exhaustion [2]. A later study dissected the subset-specific roles of mTORC1 and demonstrated that compared to Tex or effector cells, progenitor Tex cells showed reduced mTORC1 activity due to selective and heightened TGF-β signaling, but the mTORC1 pathway was reactivated after antigen stimulation, which allowed these cells to acquire a certain level of metabolic fitness [213, 214]. Treatment with rapamycin in the early stages of chronic infection led to a higher number of progenitor Tex cells (marked by TCF-1 and CXCR5 expression), fewer Tex cells (marked by TIM-3 expression), and increased proliferation as well as cytokine production, ultimately leading to enhanced systemic efficacy of anti-PD-L1 therapy [214, 215]. Treatment with rapamycin after the establishment of chronic infection was deleterious because the transition of progenitor Tex cells to effector cells (marked by CX_3_CR1 expression) required the action of the mTORC1 signaling pathway [215], which may explain the molecular similarities between CX_3_CR1^+^ effector cells and SLECs. In general, among CD8^+^ T-cell subsets, mTORC1/2 exert distinct influences through multiple regulatory mechanisms (Fig. 2).

### Sin1/mTORC2 controls T-cell memory formation and exhaustion

Adaptive immune cells, especially T cells, undergo metabolic network remodeling to initiate rapid and effective immune responses after pathogen invasion or during tumorigenesis to prevent autoimmune responses and malignancy. This process requires both agile sensors and powerful integrators, such as mTOR, which has been shown to promote T-cell development. The mTOR component Sin1 is essential for multiple stages of functional T-cell maturation from development, cytokine production and immune niche regulation. Sin1 has been shown to modulate the number of Foxp3^+^Helios^+^ thymic regulatory T cells [26]. Another study revealed a key role for Sin1 in regulating double-negative thymocyte glucose metabolism upon β-selection activation via the Akt/PPARγ signaling cascade [24]. After T cells have finished undergoing developmental processes and have egressed into tissues and the circulatory system, Sin1/mTORC2 contributes to CXCR4 expression suppression downstream of Akt/FOXO, which prevents naïve T cells from homing to bone marrow [25].

During effector and memory formation, mTORC2 has minimal impact on the effector T-cell response, but deficiency of the mTORC2 component Rictor leads to an increased potential to differentiate into memory precursors rather than SLECs [208, 216]. Interestingly, memory T cells have been found to accumulate in bone marrow. This process is controlled by CXCR4-CXCL12 and S1P-S1P. Bone marrow homing of CD8^+^ T cells facilitates antibacterial immunity after diet restriction via the downregulation of both mTORC1 and mTORC2 signaling [217], which perfectly matches our previous finding showing Sin1/mTORC2 suppression of CXCR4 prevented T-cell bone marrow accumulation.

**Fig. 4:**
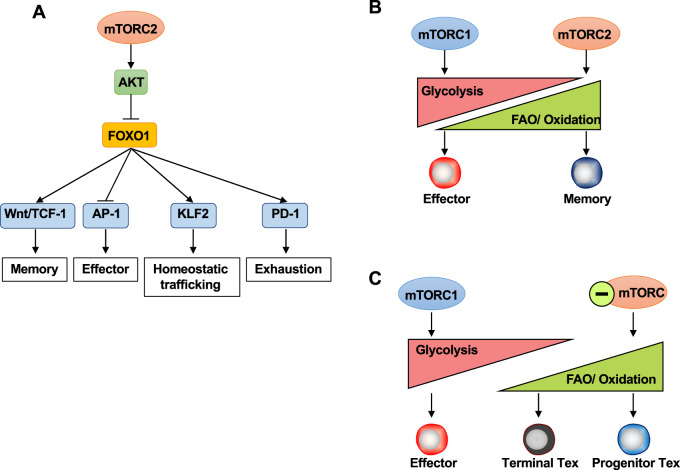
The role of the mTOR signaling pathway in metabolic programs and differentiation of CD8 T-cell subsets. **A** The mTORC2-Akt pathway promotes FOXO1 phosphorylation, resulting in decreased nuclear accumulation of FOXO1. Nuclear FOXO1 promotes memory formation through the Wnt/TCF1 pathways, directly binds and suppresses AP-1 transcription factors that are known to be key regulators of effector programs, induces KLF2 expression to regulate homeostatic trafficking, and sustains PD-1 expression while inducing the terminal differentiation of exhausted CD8^+^ T cells during chronic infection. **B** Naïve T cells uptake low levels of glucose and amino acids and rely on mitochondrial oxidative phosphorylation (OXPHOS). Upon T-cell activation, effector CD8^+^ T cells require high levels of glucose metabolism to support their rapid proliferation and production of cytokines and cytotoxic molecules. During an immune response, effector cells undergo a metabolic switch from OXPHOS to glycolysis. In contrast, memory CD8^+^ T cells have a more quiescent metabolism and rely more on OXPHOS for energy production. Memory cells also exhibit higher levels of fatty acid oxidation. mTORC1 activity is required to sustain high levels of glycolysis in effector T cells in both acute and chronic infections. Inhibition of mTORC2 activity, on the other hand, enhances the metabolic capacity of CD8^+^ T cells. **C** Tex have been reported to exhibit metabolic insufficiency with suppressed oxidation and glycolysis. Early progenitor Tex cells exhibit and retain a catabolic metabolism characterized by mitochondrial fatty acid oxidation (FAO) and oxidation

In the process of T-cell exhaustion, inhibition of the mTORC2-Akt axis leads to the translocation of FOXO1 into the nucleus, which directly regulates the expression of PD-1 and causes the terminal differentiation of PD-1^hi^ Eomes^hi^ CD8^+^ T cells (presumably Tex cells) during chronic infection [213]. A recent article revealed a positive correlation between SGK1 expression and T-cell exhaustion features in hepatocellular carcinoma [218] and suggested the possibility that the mTORC2-AKT-FOXO1 and mTORC2-SGK1 pathways are important regulatory axes for T-cell exhaustion. Additional research is needed to explore the mechanisms by which mTORC2 and Sin1/mTORC2 regulate T-cell exhaustion. The potential explanation for the diverse influences of mTORC2 on CD8^+^ T cells may be partially explained by the multiple molecules downstream of mTORC2 contributing to distinct aspects of T-cell development (Fig. 4A).

### mTOR determines the fate of CD8^+^ T cells by regulating the expression of TFs

#### T-bet and Eomes

T-bet and Eomes both belong to the T-box TF family. During acute infection, T-bet drives CTL differentiation, while Eomes promotes immunological memory [114]. IL-12 has been reported to favor effector vs. memory cell generation by promoting T-bet and inhibiting Eomes expression [219]. Furthermore, IL-12-induced STAT4 signaling pathways enhance and maintain mTOR activity, which is required to sustain T-bet expression and effector cell differentiation [204]. Rapamycin treatment upregulates Eomes expression and inhibits IFN-γ production in the secondary but not the primary phase of antigen stimulation mainly due to its role in blocking persistent T-bet expression [204]. However, the ability of mTOR to sustain T-bet expression deserves further study. In CD4^+^ T cells, mTORC1 has been reported to regulate T-bet phosphorylation to promote Th1 cell differentiation [220]. Whether this increased expression of Eomes is directly regulated by mTOR signaling pathways or is a consequence of decreased T-bet expression remains unclear.

During chronic infection, the action of the TFs T-bet and Eomes are both needed to maintain the antigen-specific T-cell pool [95]. Studies have shown that persistent T-bet expression is required for the generation of CD8^+^ T cells with higher effector function after chronic antigen stimulation, that of CX_3_CR1^+^ effector cells [3, 12, 13, 99]. Enforced expression of T-bet promoted progenitor Tex cells to differentiate into effector cells and not exhausted cells [12, 99]. mTOR activity is suppressed in progenitor Tex cells, which retain the ability to activate this pathway when differentiating into effector or Tex cells [221]. However, Eomes plays a more complex role in regulating effector- and exhaustion-related gene expression; notably, although it function together with T-bet for functional effector cell differentiation, it is expressed the highest in terminally exhausted cells and promotes T-cell exhaustion in the contexts of chronic infection and cancer [13, 95, 115]. Moreover, how mTOR controls T-bet and Eomes expression to regulate T-cell exhaustion is not fully understood.

#### FOXO1

Previous studies have shown that FOXO1 promotes memory CD8 T-cell formation by directly regulating the expression of Eomes, T-bet, and TCF-1 [222–224]. Depletion of FOXO1 reverses the effects of Rictor deficiency on the formation of memory T cells, indicating that the mTORC2-Akt-FOXO1 signaling pathway plays a vital role in controlling the differentiation of memory T cells [208]. The primary mechanism underlying transcriptional reprogramming is the nuclear stabilization of FOXO1 [208]. However, persistent antigen exposure suppresses the TCR-mediated activation of Akt and mTOR signaling [213]. mTORC2–Akt axis dysfunction leads to a decreased FOXO1 phosphorylation rate, thereby driving nuclear accumulation of FOXO1, which sustains the expression of the inhibitory receptor PD-1 and induces the terminal differentiation of exhausted CD8^+^ T cells during chronic infection [213]. Given the importance of FOXO1 in the generation of both memory and terminally exhausted T cells, further studies are needed to elucidate the underlying mechanisms involved these distinct outcomes. The following hypotheses may be tested. First, PD-1 expression is crucial for T-cell exhaustion, but several studies have indicated that in the acute infection context, PD-1 expression is required for optimal memory T-cell development [83, 225]. Thus, the Foxo1-PD-1 axis might be important for both exhausted and memory T-cell formation. Second, many TFs are common to different subsets of T cells, but they exhibit different behaviors, which are influenced by the openness of chromatin, DNA-binding partners, etc. [226]. Therefore, although different subsets of T cells are influenced by the genetic loss of FOXO1, they may also be affected by distinct epigenetic programs.

#### MYC

In T cells, MYC expression is rapidly induced in response to engagement of TCRs, and MYC expression is sustained via the actions of costimulatory receptors and cytokines such as IL-2. MYC-deficient T cells exhibit defects in glucose and glutamine metabolism and cannot proliferate or differentiate [131, 227]. During the first division of an activated CD8^+^ T-cell, asymmetric distribution of mTORC1 and amino acids leads to asymmetric MYC levels in daughter T cells [228]. Thus, daughter T cells that carry a higher level of MYC tend to follow a developmental path toward SLEC differentiation, while those with low MYC levels are more likely to differentiate into MPECs [228]. MYC expression affects other signaling pathways. For example, recently showed that activation of mTORC1 is not required for MYC expression in activated T cells, but it exerts a substantive effect on the expression of glucose transporter proteins [229].

### The interplay between differential mTORC1/mTORC2 activities and metabolic pathways in CD8^+^ T cells

The differential activities of mTORC1/mTORC2 in each CD8^+^ T subset is reflected by the distinct metabolic pathways they influence. In general, effector CD8^+^ T cells primarily undergo aerobic glycolysis, whereas memory CD8^+^ T cells show enhanced OXPHOS and fatty acid oxidation activity [132]. Moreover, the major metabolic pathways, including glycolysis and OXPHOS, in exhausted CD8^+^ T cells are severely impaired, although the exact nature of the deficiency depends partly on the type of disease [150].

mTORC1 signals are necessary for initiating the cell cycle and synchronizing the initial metabolic alterations that take place after T-cell activation. Raptor-deficient T cells cannot upregulate the expression of Glut1 or other glycolytic enzymes during their own activation and thus the production of new lipids and OXPHOS function is impaired [130]. In addition to its role in T-cell activation, mTORC1 regulates the metabolism of effector T-cell differentiation. Loss of mTORC1 leads to the upregulated expression of several transcripts related to mitochondrial metabolism, including fatty acid oxidation-related transcripts [206]. CD8 memory T cells deficient in Rictor, an essential component of mTORC2, may enhance overall metabolic fitness, as shown by the enhanced glycolysis and the SRC of mitochondria [206, 208] (Fig. 4B).

Memory T cells need to sustain effector metabolic programs after restimulation, which requires mTOR signaling. During the recall response, after TCR and costimulation, the higher activity of the mTORC2 signaling pathway within Tem and Tcm cells subsets (compared with the naïve T-cell subset) may enhance immediate glycolytic flux by activating the AKT-GAPDH axis, which allows these cells to rapidly upregulate IFN-gamma expression through histone remodeling [230]. The activation of the mTORC2-Akt signaling pathway under this condition was later shown to mediate metabolic influx in mitochondria by facilitating the binding of HK-I and VDAC [231].

mTORC1 signaling, presumably caused by persistent antigen stimulation, is thought to give rise to mitochondrial dysfunction since treatment with rapamycin largely restores most of the mitochondrial defects seen in Tex cells in the early stage of chronic infection [152]. This result may partially explain progenitor Tex cell expansion after rapamycin treatment and further suggests a promising strategy to reinvigorate Tex-cell function by manipulating mTOR-related metabolism (Fig. 4C).

### The potential of manipulating mTORC1/mTORC2 activity with pharmaceuticals

Because of the important regulatory functions of mTOR in cell growth and metabolic control, three generations of mTOR inhibitors have been developed [232]. The first generation of mTOR inhibitors consists mainly of rapamycin and its derivatives (known as Rapalogs), which target mTOR and FKBP12 to change the conformation of mTOR and thus inhibit the kinase activity of mTORC1 [232]. The second generation of mTOR inhibitors are ATP-competitive inhibitors, including TORIN, which inhibit mTORC1 and mTORC2 simultaneously [232]. Third-generation mTOR inhibitors include the Rictor-mTOR interaction blocker JR-AB2-011 and the linked Rapalog-ATP competitor RapaLink-1, which both show increased target selectivity or effectiveness [232].

Consistent with the roles of mTORC1/mTORC2 in dictating CD8^+^ T-cell fate, manipulating these pathways has been reported to endow CD8^+^ T cells with greater antitumor ability: pretreatment of CAR-T cells with rapamycin during the ex vivo expansion stage reduced mTORC1 activity and upregulated the expression of CXCR4, allowing these cells to infiltrate bone marrow and eliminate resident AML cells [233]. Another study confirmed the therapeutic potential of rapamycin in CAR-T therapy by showing that the combined use of IL-2 and rapamycin allowed human CAR-T cells to acquire memory stem cell features (CD45RA^+^CCR7^+^; CD62L^+^CD127^+^; CD62L^+^CD27^+^) [234]. Considering the effects of the mTORC2 signaling pathway, cholesterol-lowering therapy may dampen mTORC2 activity in CD8^+^ T cells, allowing these cells to acquire features of Tcm cells (higher CD62L^+^CD44^+^, Eomes^+^ cell subsets and better recall responses) and ultimately show better control over tumors [235].

Targeting mTOR signaling has been suggested to improve the efficacy of PD-1-targeted therapy. PD-1-targeted therapies enhance the T-cell response against chronic infection and tumors by promoting progenitor Tex cell differentiation into effector cell subsets [5, 9, 13]. Studies have evaluated whether an enhanced number and quality of progenitor Tex cells induced by rapamycin exerts a beneficial effect on PD-1-targeted therapy. Blocking mTOR during the T-cell expansion phase enhanced the T-cell response by inducing the accumulation of stem-like T cells, leading to increased efficacy of PD-1 immunotherapy, whereas after exhaustion had progressed, mTOR inhibition caused immunosuppression, characterized by a decrease in the number of TIM3^+^ cells and increased viral load with minimal changes to stem-like T cells [215]. Metabolically, PD-1 signals are necessary for regulating the critical balance of mTOR-dependent anabolic glycolysis and fatty acid oxidation programs to meet the bioenergetic needs of quiescent CD8^+^ memory T cells [236].

## Concluding remarks and future directions

Immunological memory and exhaustion are essential components of adaptive immunity. Our understanding of the heterogeneity and diversity of memory and exhaustion CD8^+^ T-cell subsets has increased profoundly in the past decade. However, further studies are needed to elucidate the intracellular molecules and pathways that regulate CD8^+^ T-cell fate and lead to the heterogeneity in memory T cells and Tex cells. mTOR signaling is a central hub that coordinates multiple signaling pathways and plays a critical role in regulating various aspects of T-cell function. Despite the aforementioned findings, many fundamental questions remain unanswered. For instance, how is mTOR activity precisely regulated in CD8^+^ T-cell subsets? This remains an exciting field of research. Additional research is necessary to explore the distinct roles of mTORC1 and mTORC2 in regulating the development of memory T cells and Tex cells, especially the mechanism by which mTORC2- and Sin1-mediated mTORC2 signaling leads to T-cell exhaustion. Recent studies have shown that the mTOR pathway can epigenetically regulate T cells by modulating the activity of enzymes that modify chromatin structure and DNA methylation, which in turn affects gene expression. Further research is needed to fully understand the mechanisms underlying this regulatory program and its implications for T-cell function in health and disease. CD8^+^ T-cell subsets, such as memory and effector cell subsets, exhibit distinct metabolic requirements, and meeting these needs is critical for their function and survival. Over several decades of research, mTOR has been identified as a key regulator of multiple metabolic processes. It controls the overall level or functional activity of multiple metabolic enzymes by sensing alterations in metabolite levels, especially in glucose and amino acid levels. The molecular mechanisms by which mTOR signaling regulate glycolytic pathways, oxidation and mitochondrial function in T cells are also incompletely understood. Additional research is required to clarify the potential of targeting the mTOR pathway as a therapeutic strategy for modulating cellular metabolism and enhancing the restoration of exhausted T-cell function.
