# Author Correction: Regulation of CD8^+^ T memory and exhaustion by the mTOR signals

**DOI:** 10.1038/s41423-023-01090-1

**Published:** 2023-10-11

**Authors:** Yao Chen, Ziyang Xu, Hongxiang Sun, Xinxing Ouyang, Yuheng Han, Haihui Yu, Ningbo Wu, Yiting Xie, Bing Su

**Affiliations:** 1 Shanghai Institute of Immunology, Department of Immunology and Microbiology, and The Ministry of Education Key Laboratory of Cell Death and Differentiation, Shanghai Jiao Tong University School of Medicine, Shanghai, 200025 China; 2 Department of Tumor Biology, Shanghai Chest Hospital, Shanghai Jiao Tong University School of Medicine, Shanghai, 200025 China; 3 Center for Immune-Related Diseases at Shanghai Institute of Immunology, Department of Gastroenterology, Ruijin Hospital, Shanghai Jiao Tong University School of Medicine, Shanghai, 200025 China; 4 Shanghai Jiao Tong University School of Medicine–Yale Institute for Immune Metabolism, Shanghai Jiao Tong University School of Medicine, Shanghai, China; 5 Key Laboratory of Molecular Radiation Oncology of Hunan Province, Xiangya Hospital, Central South University, Changsha, China

**Keywords:** Signal transduction, Adaptive immunity

Correction to: *Cellular & Molecular Immunology* 10.1038/s41423-023-01064-3, published online 15 August 2023

In this article the author name Yuheng Han was incorrectly written as Yuhen Han. The original article has been corrected.

